# HOME TRIAD: A NEW WAY TO UNDERSTAND THE MEANING OF HOME FOR PEOPLE LIVING WITH DEMENTIA

**DOI:** 10.1093/geroni/igad104.3393

**Published:** 2023-12-21

**Authors:** Wenjin Wang, Hao Huang

**Affiliations:** Texas A&M University, College Station, Texas, United States; Texas A&M University, College Station, Texas, United States

## Abstract

Highlighted by the frequent stating “I want to go home,” understanding the meaning of home for people living with dementia (PLWD) is crucial to provide them better care and environment. Our novel theoretical framework, termed ’home triad’, abstracts “home” into three interconnected pillars: conceived, perceived, and lived home. We conducted in-depth interviews with five PLWD, who were over 65 years old with MMSE scores between 11-24, received home care, and could understand and consent to the study. Their caregivers were also consulted for member-checking. The findings showed that the early memories of home were more vivid. The lived home experience held paramount significance; while stable housing was vital, family was of utmost importance to PLWD. Additionally, a gender-based distinction in lived home was evident. It was also found that PLWD’s perceived daily activities and lived memories expanded their home beyond their immediate residence, encompassing neighborhoods, villages, and cities. Within home triad, we noticed that the conceived home, focusing on the discourses of power and knowledge, could easily be overlooked. Yet, focusing on this pillar revealed the pivotal roles of design and caregivers in shaping PLWD’s home, which in turn influenced perceived and lived experiences. Through home triad, we can better appreciate profound home memories for PLWD. This could bolster reminiscence therapy and enable more effective home modifications, or even improve institutional homelike environments. Our future direction aims to refine and apply home triad with more home stories, thereby informing research and practice to enhance dementia-friendly environment and PLWD’s well-being.

